# Digital tools not enough; quality control–linked incentives are needed for tuberculosis active case finding

**DOI:** 10.5588/pha.26.0015

**Published:** 2026-08-24

**Authors:** H. Abdullah, H.D. Shewade, P. Manickam, K. Gupta, M.S. Ambiya, R.S. Dhakad, K. Sharma, S. Nag, M. Shahnawaz, R. Nagar

**Affiliations:** 1 Khushi Baby, Jaipur, India;; 2 Division of Health Systems Research, ICMR National Institute of Epidemiology (ICMR-NIE), Chennai, India;; 3 Department of Community Medicine, Geetanjali Institute of Medical Sciences, Jaipur, India.

**Keywords:** tuberculosis, active case finding, digital health survey, cross-verification

## Abstract

**BACKGROUND:**

Low yield of tuberculosis (TB) active case finding (ACF) in routine health systems is a concern. Following identification of high-risk villages/wards guided by annual digital household survey (captured in Community Health Integrated Platform [CHIP]), the health system utilised the ACF module of CHIP for an ACF cycle (December 2025 to January 2026) in Udaipur district, Rajasthan, India. Despite advocating the recommendations from a recently concluded national-level TB ACF evaluation, sputum collection and transport and quality control–lined incentives were not implemented.

**OBJECTIVES:**

To document the reported and estimated (after telephonic data cross-verification by investigators) scale, quality, and yield of ACF, based on a longitudinal descriptive study using secondary data.

**RESULT:**

Of 1.3 million high-risk populations, 70.6% (scale) were reportedly screened. With 44 (0.6% test positivity) reported ACF-detected TB, the number needed to screen (NNS) was 21,082 (quality) and yield was 0.7% (total ACF-detected TB divided by [0.5% × total high-risk population]). The estimated scale was 35% (n = 457,240) and one out of 44 was confirmed to be ACF-detected (estimated NNS = 457,240, estimated yield <0.1%). Similar suboptimal results were observed in 33% villages/wards that were multidimensionally poor as per CHIP and additional 21% villages/wards identified as high-risk through local mapping.

**CONCLUSION:**

Suboptimal ACF yield reveals digital tools alone are insufficient.

Despite efforts to combat tuberculosis (TB), it continues to be a global public health challenge. The WHO has set ambitious targets to end TB, but achieving these requires innovative and effective strategies.^1^ Active case finding (ACF) plays a critical role in identifying the missing TB, especially in marginalised and vulnerable populations (also called high-risk populations).^2^ Globally, India has the highest TB burden.^2^ In India, in addition to undernutrition, diabetes mellitus, tobacco, alcohol, and HIV, access to clean fuels (leading to indoor air pollution), poverty, social protection, and living in informal settlements are broader socio-economic determinants for TB.^3^ To detect the missing TB (0.2 million in India in 2024), India’s National Tuberculosis Elimination Programme (NTEP) has been implementing ACF for high-risk populations since 2017.^3,4^ ACF for TB includes systematic screening for TB among household contacts and institutional and population-based high-risk groups that are estimated to have at least 0.5% prevalence of undetected TB (see Supplementary Data Figure S1; Supplementary Data Table S1).^5^

A recent national-level evaluation of ACF in India (2022–2024) identified suboptimal scale and quality due to several limitations that undermined effectiveness **(**for six tips, see Supplementary Data Box S1).^6–8^ This included inadequate or limited mapping of high-risk populations before ACF, ad-hoc ACF activities, lack of knowledge and know-do gap, lack of provision to document ACF-detected presumptive TB (aggregate number to be captured in *Ni-kshay*, web-based patient management system of NTEP), lack of accountability mechanisms, including the limited use of quality assurance approaches linked to incentive disbursement for ACF activities (see Figure 1), and a complex ACF module in *Ni-kshay*.^6–8^ Finally, digital tools or even paper-based ACF tools were not utilised for individual-level data collection in the field. Therefore, mechanisms for cross-checking aggregate ACF care cascade data entered in *Ni-kshay* (NTEP’s case-based TB information management system) were not available or implemented.^6–8^

**FIGURE 1.**
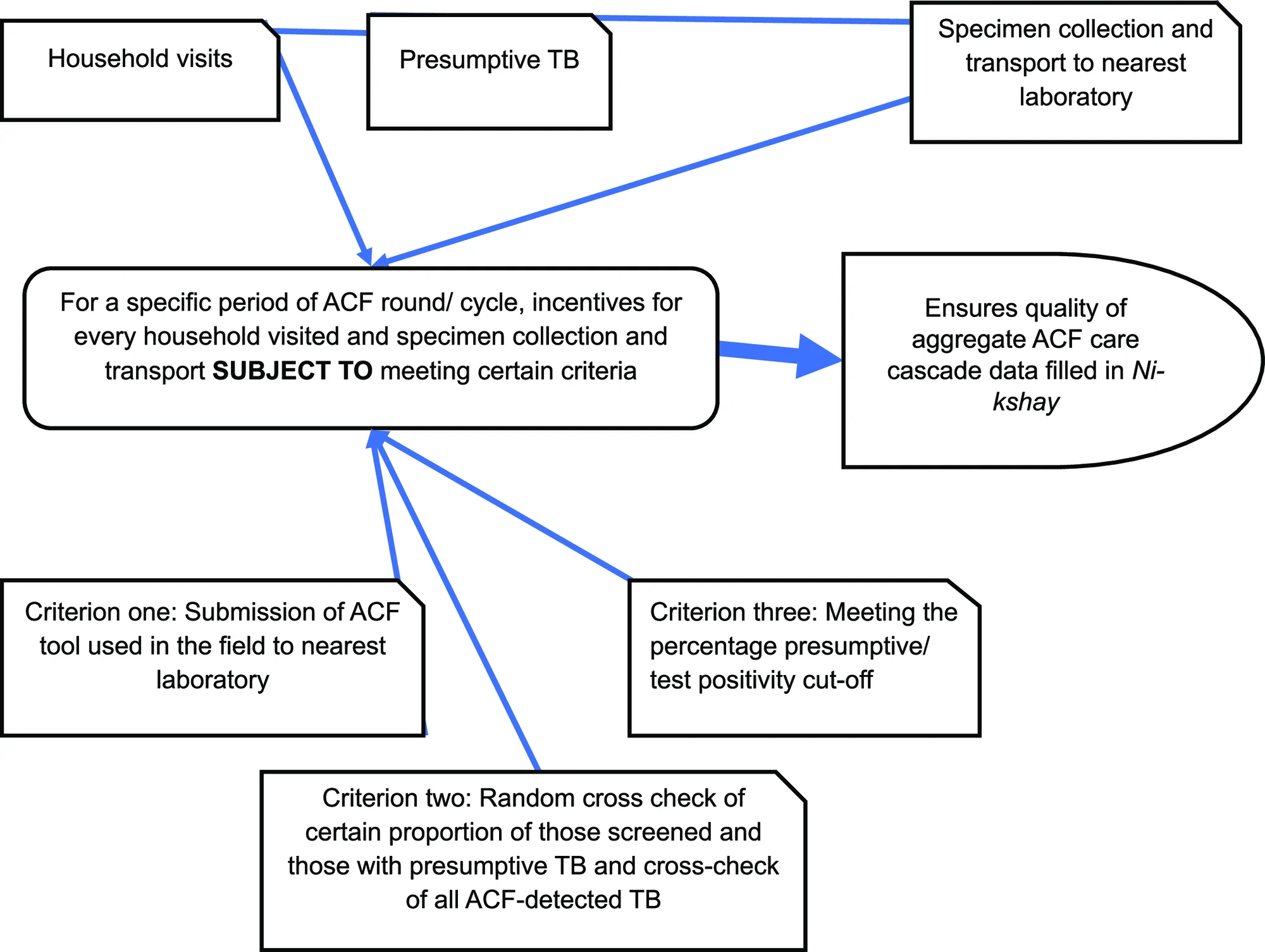
Quality control–linked incentive framework for active case finding (ACF) in TB.^7^ Reprinted after minor modifications with permission from Shewade et al.^7^ under a CC BY license. TB = tuberculosis.

Prior to ACF, local high-risk population mapping is recommended by NTEP (see Supplementary Data Table S1).^9^ While this is cumbersome, the availability of a digital annual household survey in a state like Rajasthan (northern India) provided a unique opportunity to identify high-risk villages (at state and district level) that can be leveraged for data-driven ACF. The Community Health Integrated Platform (CHIP) developed by Khushi Baby (a non-profit organisation) is being used for the village-level digital annual household survey covering over 85% of villages across 19 districts (half of the total districts) in Rajasthan.^10^ Through a mapping exercise based on digital annual household survey published by the authors, high-risk villages were identified that were multidimensionally poor, had >60% households belonging to marginalised communities, and were far away from the nearest primary health facility.^11^ Following the mapping exercise, in Udaipur district, Rajasthan, we used the next available TB ACF cycle to assess the implementation performance in these high-risk populations identified through digital annual household survey. This included ACF scale, quality, and yield across different high-risk population groups and independent (by the investigators) cross-verification of reported ACF data. The first group included high-risk villages/wards with a high multidimensional poverty index (MDPI) as per the digital annual household survey.^11^ The second group included high-risk villages/wards (high extent of individuals with TB risk factors) identified through local mapping (and not being part of first group). The third group was built based on digital annual household survey that included high-risk individuals who did not belong to first and second groups. If the ACF scale and quality indicators in the first group are found to be as good as (if not better) the second and third groups, this could provide a unique opportunity to easily prioritise limited available resources for ACF and scale this up in remaining districts. We also leveraged this opportunity for cross-verification of aggregate ACF care cascade data that are captured in the TB ACF module of CHIP. This addressed a major limitation identified in the national-level ACF evaluation.^6–8^

The aim of this operational research was to document the reported and estimated (after telephonic data cross-verification by investigators) scale, quality, and yield of ACF, during the TB ACF cycle from 25 December 2025 to 31 January 2026 in Udaipur, Rajasthan.

## METHODS

This was a longitudinal descriptive study involving secondary data.

### General setting

While the estimated prevalence of TB (2019–2021) in Rajasthan is 484 per 100,000 population, the state notifies 209 people per 100,000 population annually.^3,12^ This indicates a high prevalence-to-notification ratio (2.3:1) when compared to national estimates (1.8:1).^13^ Udaipur district, located in the southern part of Rajasthan, has 541 village *panchayats* (local government bodies) with 2,390 villages and 71 wards under the city municipal council, serving a diverse population of 3.1 million. Udaipur is supported by 121 primary health centres, 132 TB diagnostic centres under 13 TB units (sub-district-level administrative units), and 1 district TB cell.

### Digital annual household survey in Rajasthan (CHIP)

Under the national health mission, the Accredited Social Health Activists (ASHAs – honorarium-based community health activists cum workers) under the guidance of Auxiliary Nurse Midwife (stationed at the level of health subcentres, catering to a population of ≈5,000) conduct annual household survey and activities under various national health programmes.^14^ Annual household survey provides the denominators for the calculation of indicators of various programmes under the national health mission. CHIP captures comprehensive individual demographic, health, and socio-economic information of annual household survey.^10^ Data can be captured in offline mode and later synced when internet is available. CHIP has a module for TB ACF where individual-level ACF data can be captured. This provided an opportunity for random cross-verification of ACF data and implementation monitoring that aids in quality control–linked incentives (Figure 1).^6^

### TB ACF cycle in Udaipur

One TB ACF cycle (intention to cover the high-risk population once) was implemented from 25 December 2025 to 31 January 2026. The population targeted for screening included 791 (33%) high-risk villages/wards under group 1 with the highest MDPI and 506 (21%) high-risk villages under group 2. All households in these high-risk villages and high-risk individuals belonging in group 3 were to be screened. Screening was done in routine health settings using the available resources and workforce. The investigators and the district TB cell provided the necessary technical support in planning, implementation, and monitoring. The implementation was preceded by administrative orders from the state. The existing ACF algorithm of symptom screening followed by referral/sputum collection and transport (SCT) for confirmation along with prioritisation of rapid molecular diagnosis tests for ACF-detected presumptive TB was implemented (see Supplementary Data Figure S1; Figure 1). Incentives were provided: ₹10/- for each household screened and ₹150/- for SCT and ₹500/- for each ACF-detected person with TB.

Although the intention was to implement this ACF cycle in line with the national ACF guidance and recommendations of the national-level ACF evaluation (see Supplementary Data Box S1),^7,15^ there were some limitations. To ensure both quality and accountability in ACF cycle, we had planned that ASHA workers would be eligible for incentive only when the data submitted met a minimum threshold quality. Specifically, at least 5% of those screened should be identified as presumptive and SCTs done for them. Despite advocacy efforts at the state and district-level, while patient referral was done, neither SCT was systematically done nor incentives were quality control–linked (Figure 1). They were disbursed without data cross-verification and irrespective of meeting the 5% presumptive criterion. Individual-level ACF data in CHIP were regularly cross-checked during ACF by the Khushi Baby team: a certain percentage of those screened, those presumptive for whom SCT was done, and all ACF-detected TB were cross-checked through telephonic conversation. Individuals selected for telephonic verification represented a programmatic quality-assurance sample rather than a statistically representative sample. Verification findings were therefore interpreted cautiously and used to generate approximate adjusted estimates.

### Study population, variables, and sources of data

For individuals that were screened, the following variables were extracted from TB ACF module of CHIP: unique identifier, high-risk group, date of ACF activity, age, gender, presumptive TB (yes/no), date of sputum collection, date of sputum examination, TB diagnosis (yes/no), and date of treatment start.

### Data analysis

Data were analysed using Python software, and all findings were reported as frequency and proportion (except number needed to screen [NNS]). Reported scale, quality, and yield of ACF cycle were calculated; overall and stratified by age, gender, and high-risk group. The three high-risk groups were operational categories used for programme planning and implementation. They were identified using different criteria and were not intended to represent statistically comparable cohorts. Stratified analyses were therefore descriptive. Scale was defined as the extent of high-risk population screened. Quality was defined as the NNS to detect one ACF-detected TB (higher the NNS, lower the quality). Yield was defined as the estimated percentage of undetected TB in high-risk populations detected during the ACF cycle (total ACF-detected TB divided by [0.5% × total high-risk population]). The extent of cross-verification of aggregate ACF numbers at each step of the care cascade and its findings were also reported. Based on these findings, approximate adjusted estimates of scale, quality, and yield were generated to assess the potential magnitude of discrepancies in reported ACF data.

### Ethical statement

Ethics approval was obtained from the Institutional Human Ethics Committee of ICMR National Institute of Epidemiology (ICMR-NIE), Chennai, India (NIE/IHEC/A/202408-14 dated September 11 2024 and renewed on June 26 2025). As the study involved the use of district-level secondary data (electronic), waiver of written informed consent was sought and approved.

## RESULTS

Of the 1,314,598 high-risk populations, a total of 927,609 (70.6%) individuals were screened. A total of 540,292 (58.2%) belonged to high-risk group 1. The additional contribution of high-risk group 2 was 146,582 (15.8%) and of high-risk group 3 was 240,735 (25.9%) (Supplementary Data Table S2). Of those screened, 16,526 (1.8%) individuals were identified as presumptive TB and of them, 6,815 (41.2%) underwent confirmation through sputum examination. A total of 44 (0.6% test positivity) individuals were reported as ACF-detected TB. The reported NNS was 21,082, and the reported yield was 0.7% (Supplementary Data Table S1; Table).

**TABLE.** Estimated yield, scale, and quality of TB ACF cycle conducted from December 2025 to January 2026 in identified villages of Udaipur, Rajasthan, India.

Care cascade step	Reported		Estimated	
	n	%	n	%
Target population	1,314,598			
Population screened (Scale)	927,609	(71)	457,240	(35)
Presumptive TB	16,526	(2)	6,841	(1)
Tested	6,815	(41)	4,089	(60)
Diagnosed with TB	44	(<0.1)	1	(<0.1)
Number needed to screen (Quality)	21,082		457,240	
Yield	–	(0.7)		(<0.1)

TB = tuberculosis; ACF = active case finding.

Estimated values are adjusted using proportions from telephonic verification; percentages use the preceding cascade step as the denominator.

### Cross-verification of ACF care cascade aggregate numbers

**FIGURE 2.**
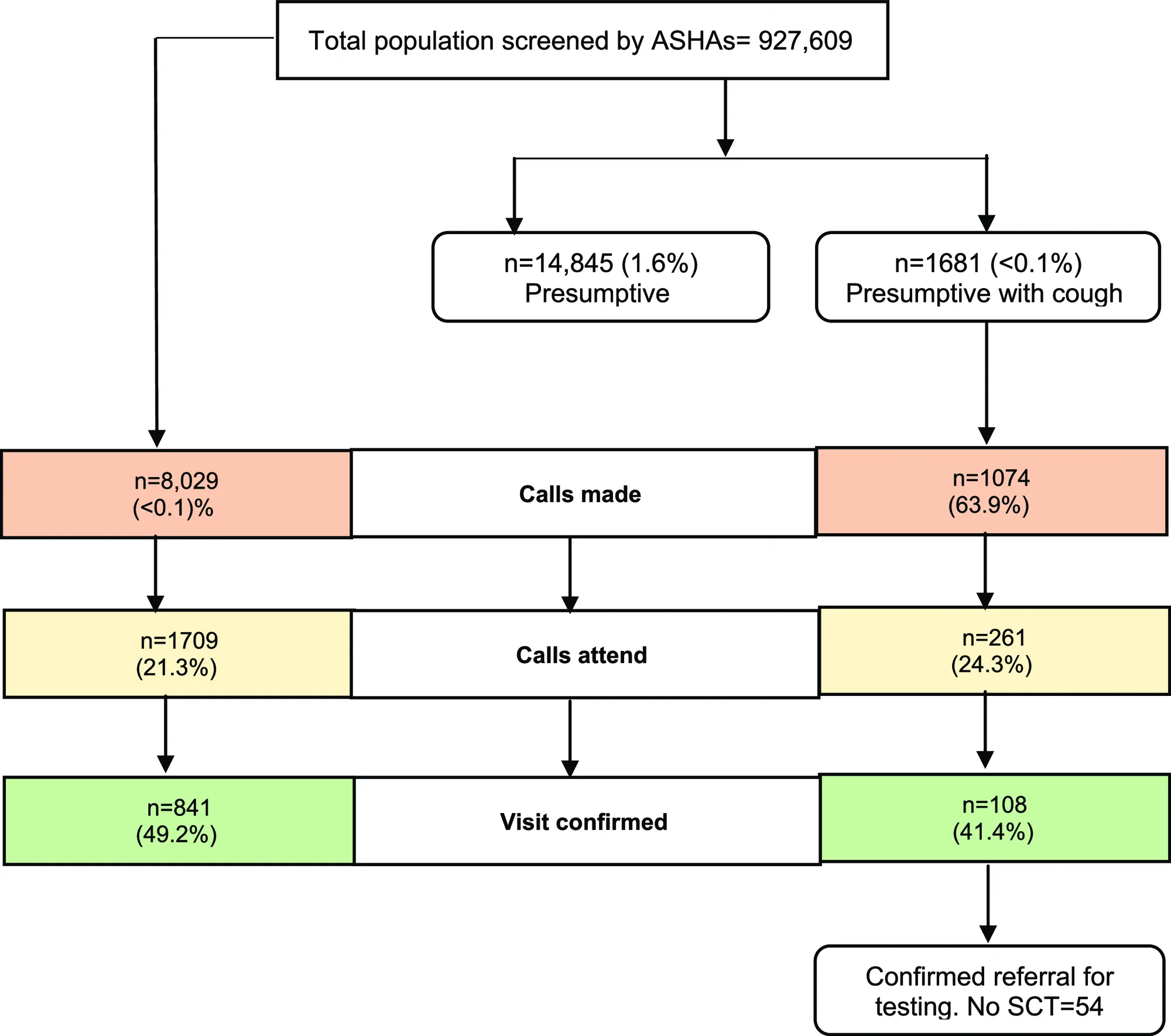
Findings of cross-verification of aggregate numbers generated in the active case finding (ACF) care cascade, December 2025 to January 2026, Udaipur, Rajasthan, India. ASHA = Accredited Social Health Activist; SCT = sputum collection and transportation.

Due to the short duration of the verification window within this period, calls could be attempted for 8,029 (1%) high-risk individuals reported as screened, of whom 1,708 (21.3%) were successfully contacted. Among those contacted, 841 (49.2%) confirmed that an ASHA had visited their home for screening (Figure 2). Among those reported as presumptive with cough symptoms (n = 1,681), calls were placed to 1,074 (64%) individuals, and 261 (24.3%) were successfully contacted. Of these, 108 (41.4%) reported home visit by an ASHA for screening; of whom, 54 (50%) were referred to the nearest hospital or health centre for testing (Figure 2). None underwent SCT. Of the 44 reported as ACF-detected TB, 1 was confirmed to be detected because of this ACF cycle; the remaining 43 were detected through passive case finding or were already on treatment. The estimated yield, scale, and quality of TB ACF after considering cross-verification findings are depicted in the Table.

## DISCUSSION

Across all three high-risk groups, the scale, quality, and yield were substantially poor, reflecting persisting structural issues in TB ACF implementation in line with findings of the national-level evaluation.^6,7,15^ Cross-verification revealed major discrepancies, raising concerns about the reliability of aggregate numbers reported in ACF cascade.^6^ Almost all individuals reported as ACF-detected TB could not be directly attributed to be detected through ACF during the ACF cycle. Only one ACF-detected TB (post cross-verification) was identified in the whole district of Udaipur. A key strength of this study was the use of digitally captured annual household survey data (routinely captured in the state), which enabled identification of high-risk villages. Digitally captured ACF care cascade data facilitated cross-verification.

There were some limitations. The study relied on secondary data reported by ASHAs, which may be subject to data entry or reporting errors. Certain external factors, such as incentive structures influencing the quantity and quality of reporting, were not examined systematically as they were beyond the scope of the study. Telephonic verification was subject to selection bias, non-response bias, and recall bias. In addition, the verification sample was intended for programmatic quality assurance and may not have been representative of all screened individuals. Therefore, adjusted estimates should be interpreted as indicative rather than precise population estimates. This study was operational and descriptive in nature and was not designed to establish causal relationships. The findings reflect implementation experience from a routine programme setting and should therefore be interpreted as implementation evidence rather than definitive evidence regarding the effectiveness of digital tools, incentive structures, or specific ACF approaches.

Limitations notwithstanding, there were key policy-relevant findings. The observed findings of highly suboptimal quality and yield may be explained by the lack of use of quality control–linked incentives, lack of implementation monitoring backed by acting on the findings of cross-verification (which was investigator-led), overreliance on referral (not implementing SCT systematically), and not following the six tips for implementing TB ACF that were disseminated to all the states following the national-level evaluation.^6,7,15^ This is despite multiple efforts of advocacy with the state before this TB ACF cycle and published evidence in this regard over the past many years.^6,7,15,16^ To put it simply, if ASHAs get ₹10 per household visited, they will be eligible for ₹2,000 if they just report screening of 200 households in the TB ACF module of CHIP. If the incentive is released as such without any cross-verification and quality control (Figure 1), then the ASHAs may not take the efforts to truly screen, identify presumptive TB, and do SCT to the nearest TB diagnosis centre to claim ₹150 per SCT. In addition, anecdotally, inadequate and inconsistent training of ASHAs might have further contributed to knowledge and know-do gaps.

Screening many people does not always lead to finding more TB cases when testing and quality control are weak. Programmatic attention appears to be more towards achieving numerical targets rather than ensuring quality. This was also noted in the national-level evaluation of TB ACF, where the scale and quality of TB ACF was way poorer when scale and quality were documented based on aggregate numbers generated through cross-verifiable individual-level ACF screening data than what is being programmatically reported.^7,15^

The study has key implications. It demonstrates that digital tools alone are insufficient unless accompanied by systematic training, cross-verification, diagnostic linkage through SCT, and accountability mechanisms.^6^ Incorporating routine verification of reported ACF data and linking incentives to quality indicators may improve the reliability and effectiveness of ACF programmes. This could be considered as part of routine monitoring by Rajasthan NTEP. To complement monitoring by Rajasthan NTEP, Independent monitoring may be considered in future implementation efforts.

Considering the limited bandwidth of the existing health system, implementation of TB ACF may be handed over to non-governmental organisations in health (not-for-profit) with the state and district TB cell engaged in quality control, cross-verification, and monitoring (e.g., Project Axshya).^16–20^ If TB ACF implementation is continued by the health system utilising existing workforce, it should be guided by the six tips to conduct TB ACF (see Supplementary Data Box S1). Pilots should be implemented at a small scale to first improve our understanding, and based on these learnings, TB ACF should be scaled up. Based on local context and resources, alternate case-finding strategies may also be considered, like i) systematic passive case finding in health facilities along with SCT for those who undergo pre-testing loss to follow-up and ii) private pharmacy–based TB surveillance.^21^ This should be guided by operational research.

## CONCLUSION

The TB ACF cycle in Udaipur demonstrated there are substantial discrepancies between reported and verified scale, quality, and yield. Although digital tools facilitated data capture and cross-verification (investigator-led), additional measures such as stronger implementation monitoring, programme-led data cross-verification, and improved accountability mechanisms may be required to improve ACF scale, quality, and yield. This should be guided by operational research. Further operational research is needed to identify effective strategies for improving the quality and effectiveness of routine ACF activities.

## Supplementary Material
