# Supplementary material for: Digital tools not enough; quality control–linked incentives are needed for tuberculosis active case finding
Source: Public Health Action. 2026 Aug 24;16(3):183–7. doi: 10.5588/pha.26.0015 (PMC13502500; doi:10.5588/pha.26.0015)
Supplement: Supplementary file 1 [file pha26-0015_supplementarydata1.pdf]

Supplementary Box I. Six tips for implementing TB ACF: Recommendations of ICMR-NIE-led national-level TB ACF evaluation project (2022-24) [7]

1. Planning: Map high-risk populations, decide number of cycles, mode of ACF cycle, PIP, Microplanning, volunteer list
2. Training: to fill the knowledge gaps, tailored to type of staff, clarify incentive quality control criteria
3. Use of ACF tool in the field and mechanisms for regular cross-checking of ACF activities
4. Quality control-linked incentives
5. Simplifying ACF module in *Ni-kshay* for data entry and export
6. Integrated ACF

TB: tuberculosis; ACF: Active case finding; ICMR-NIE: Indian Council of Medical Research-National Institute of Epidemiology

Supplementary Table 1. Broad high-risk groups for active case finding for TB [15]

Clinical	Socioeconomic	Geographical
• Contacts of TB patients<br>• Undernutrition<br>• HIV<br>• High risk groups for HIV identified by the program<br>• Noncommunicable diseases [Diabetes, hypertension, other cardiovascular diseases, chronic lung/liver/renal disease and their risk factors (alcohol, smoking, drug addiction, others)]<br>• COVID19 recovered patients<br>• Immunocompromised (cancer), on Palliative care<br>• Occupational risk with TB (construction site workers, weaving/glass industry workers, miners, cotton mill workers, stone crushers, beedi rolling, poultry workers, tea garden workers, health care workers)<br>• Elderly	• Prisons<br>• Old age homes, night shelters, home for destitute, orphanages, refugee camps, asylums<br>• Street children / homeless<br>• Unorganized labour<br>• Migrant population<br>• Slums<br>• Areas with high level of poverty / people living the below poverty line<br>• Populations known to drink raw milk/uncooked meat<br>• Indigenous/ tribal population<br>• Sanitary workers	• Hard to reach areas<br>• Villages seeking care from traditional healers<br>• Areas with clustering of cases<br>• Areas with low notification (than expected)

Abbreviations: TB, tuberculosis; HIV, human immunodeficiency virus; COVID19, corona virus disease 2019

[a]these specific high-risk groups may also be classified into facility-based and population-based, facility-based included orphanages, prison, refugee camps, homes for the destitute, tribal schools homes, old age homes, night shelters, construction site workers, cotton mill workers, mine workers, stone crushers, tea garden workers, weaving, glass workers, industrial workers, poultry workers, beedi workers

Reprinted with permission from Shewade HD et al [15]under a CC BY license

Supplementary Figure 1. Scale, quality and yield* of active case finding (ACF) for tuberculosis (TB)in high-risk populations, India.[13]

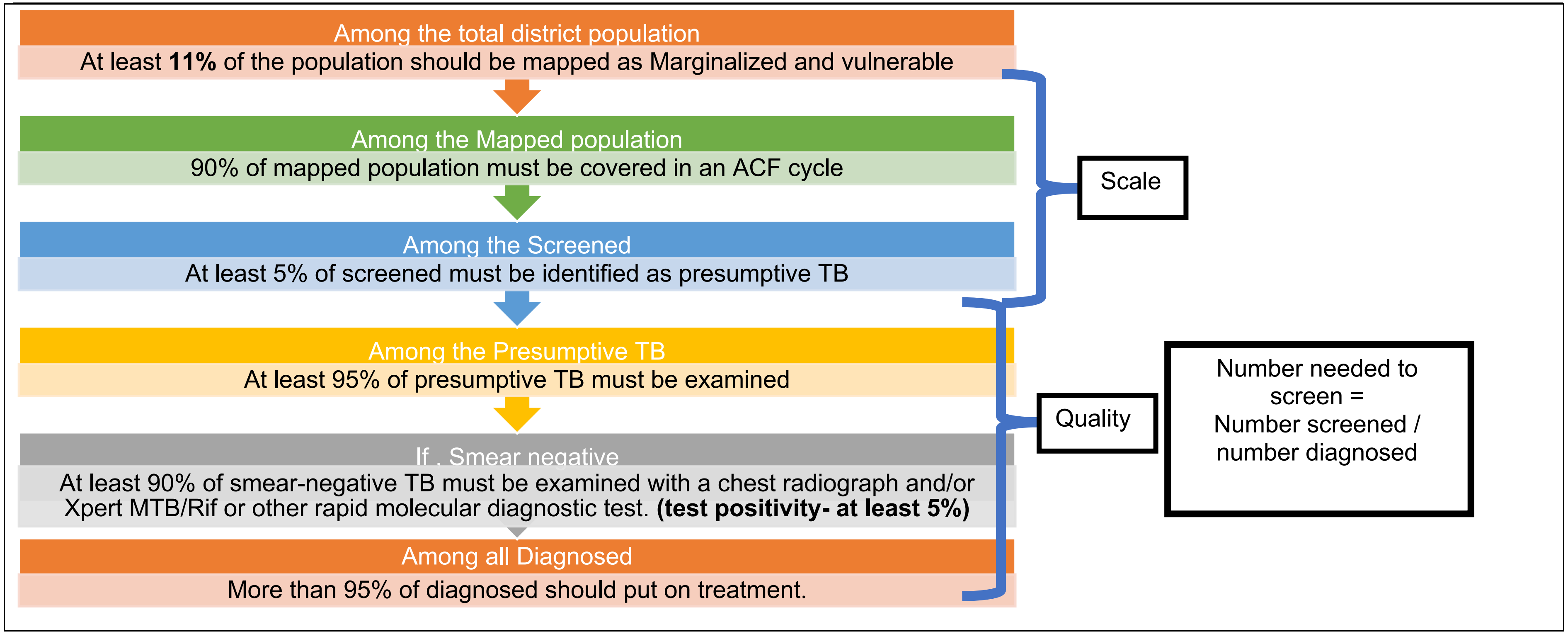

*Yield of an ACF cycle = Total detected because of ACF in high-risk populations / Total estimated undetected TB in the high-risk populations

Supplementary Table 2. Quality* indicators in the TB ACF cycle conducted from December 2025 to January 2026 in identified villages of Udaipur, Rajasthan, India

**Category**		**Screened**		**Presumptive**		**Tested**		**Diagnosed**		**NNS***
		**n**	**(%)**	**n**	**(%)**	**N**	**(%)**	**n**	**(%)**	
**Total**		**9,27,609**	**(71)**	**16,526**	**(1.8)**	**6,815**	**(41.2)**	**44**	**(0.6)**	**21,082**
Gender	Male	4,94,590	(53.3)	8,283	(1.6)	3,485	(42)	35	(1.0)	14,131
	Female	4,32,755	(46.6)	8,240	(1.9)	3,330	(40.4)	9	(0.3)	48,083
	Other	264	(0.03)	3	(1.1)	0	(0)	0	(0)	NA
Age in yrs	≥60 (elderly)	1,91,366	(20.6)	5,865	(3.0)	2,402	(40.9)	11	(0.5)	17,396
	>60	7,36,243	(79.3)	10,661	(1.4)	4,413	(41.3)	33	(0.7)	22,310
Type of high-risk population										
	Group 1: Multidimensionally poor villages - CHIP	5,40,292	(58.2)	6,205	(1.1)	1,133	(18.2)	7	(0.6)	77,184
	Group 2: High-risk villages - local mapping (not in group1)	1,46,582	(15.8)	5,382	(3.6)	1,690	(31.4)	22	(1.3)	1,094
	Group 3: High-risk individuals - CHIP (not in group 1 or 2)	2,40,735	(25.9)	4,939	(2.0)	3,992	(80.8)	15	(0.4)	9,772

TB: tuberculosis; ACF: active case-finding; CHIP: Community health integrated platform; NNS: number needed to screen; *The estimated scale, quality after accounting for the finding of cross-verification are presented in table 1
